# Cutaneous sarcoidosis due to immune‐checkpoint inhibition and exacerbated by a novel BRAF dimerization inhibitor

**DOI:** 10.1002/ski2.71

**Published:** 2021-10-20

**Authors:** J. P. Pham, P. Star, S. Wong, D. L. Damian, R. P. M. Saw, M. J. Whitfeld, A. M. Menzies, A. M. Joshua, A. Smith

**Affiliations:** ^1^ St Vincent's Hospital Sydney New South Wales Australia; ^2^ St Vincent's Clinical School University of New South Wales Darlinghurst New South Wales Australia; ^3^ Melanoma Institute of Australia The University of Sydney Sydney New South Wales Australia; ^4^ Faculty of Medicine and Health The University of Sydney Sydney New South Wales Australia; ^5^ Royal Prince Alfred Hospital Camperdown New South Wales Australia; ^6^ The Skin Hospital Darlinghurst New South Wales Australia; ^7^ Royal North Shore and Mater Hospitals Sydney New South Wales Australia

## Abstract

Sarcoidosis is a non‐infective granulomatous disorder of unknown aetiology, with cutaneous involvement affecting up to 30% of patients. Drug‐induced sarcoidosis has been reported secondary to modern melanoma therapies including immune‐checkpoint inhibitors and first generation BRAF inhibitors such as vemurafenib and dabrafenib. Herein, we report a case of cutaneous micropapular sarcoidosis that first developed on immune‐checkpoint inhibition with ipilimumab and nivolumab for metastatic melanoma, which was exacerbated and further complicated by pityriasis rubra pilaris‐like palmar plaques upon transition to a next‐generation BRAF‐dimerisation inhibitor. Both the micropapular eruption and palmar plaques rapidly resolved after cessation of the novel BRAF‐inhibitor and concurrent commencement of hydroxychloroquine. It is unclear how inhibition of BRAF‐dimerisation results in granuloma formation, though upregulation of T_H_1/T_H_17 T‐cells and impairment of T‐reg cells may be responsible. Clinicians should be aware of the potential for exacerbation of sarcoidosis when transitioning from immune‐checkpoint inhibitors to these novel BRAF‐dimerisation inhibitors, particularly as their uptake in treating cancers increases beyond clinical trials. Further studies are required to assess whether these next‐generation agents can trigger sarcoidosis de‐novo, or simply exacerbate pre‐existing sarcoidosis.

1

**What's already known about this topic?**

- Immune‐checkpoint inhibition and first generation BRAF inhibitors can trigger sarcoidosis when used to treat metastatic melanoma.

**What does this study add?**

- Immunotherapy associated sarcoidosis can be exacerbated by inhibition of BRAF‐dimerisation.
- Novel BRAF‐dimerisation inhibitors may also precipitate psoriasiform, pityriasis rubra pilaris‐like palmar plaques.
- Hydroxychloroquine can be an effective treatment for the cutaneous sarcoidosis, with additional anti‐neoplastic properties.

## 2 INTRODUCTION

Sarcoidosis has been reported secondary to both immune‐checkpoint inhibitors (ICI's) and first‐generation *BRAF*‐inhibitors, however the pathogenesis of these granulomatous reactions is unknown.^1^, ^2^ Herein, we present a case of cutaneous sarcoidosis that first developed on immune‐checkpoint inhibition, which was exacerbated and further complicated by palmar psoriasiform plaques upon transition to a next‐generation *BRAF*‐dimerisation inhibitor.

## 3 CASE REPORT

A 36‐year‐old man was diagnosed with acral lentiginous melanoma of his left 4^th^ toe (Breslow depth 9.3 mm) in December 2018, with in‐transit cutaneous metastases on his left leg and abdominal wall (*American Joint Committee on Cancer 8* ^*th*^ *Edition*‐stage M1a). His medical history was notable for asthma and prior psoriasis limited to scalp and inguinal regions. After surgical resection (*NRAS‐Q61K* mutation confirmed on sequencing), adjuvant nivolumab was commenced. New inguinal lymph node disease in November 2019 required addition of ipilimumab with a complete response in the inguinal node, but no response in the cutaneous metastases (necessitating further topical diphencyprone immunotherapy). Immune hepatitis and hypophysitis required cessation of ipilimumab after three cycles, and addition of oral prednisolone.

In July 2020 he developed micropapules involving the axillae and inguinal creases, diagnosed as cutaneous sarcoidosis on biopsy. Serum angiotensin converting enzyme (ACE) was elevated 1.2 times the upper limit of normal, with multi‐system reviews negative for systemic sarcoidal involvement. As the cutaneous sarcoidosis was asymptomatic, nivolumab was continued. Progressive cutaneous metastases to his left leg and arm precipitated transition onto a clinical trial of a novel *BRAF*‐dimerisation inhibitor in January 2021.

**FIGURE 1:**
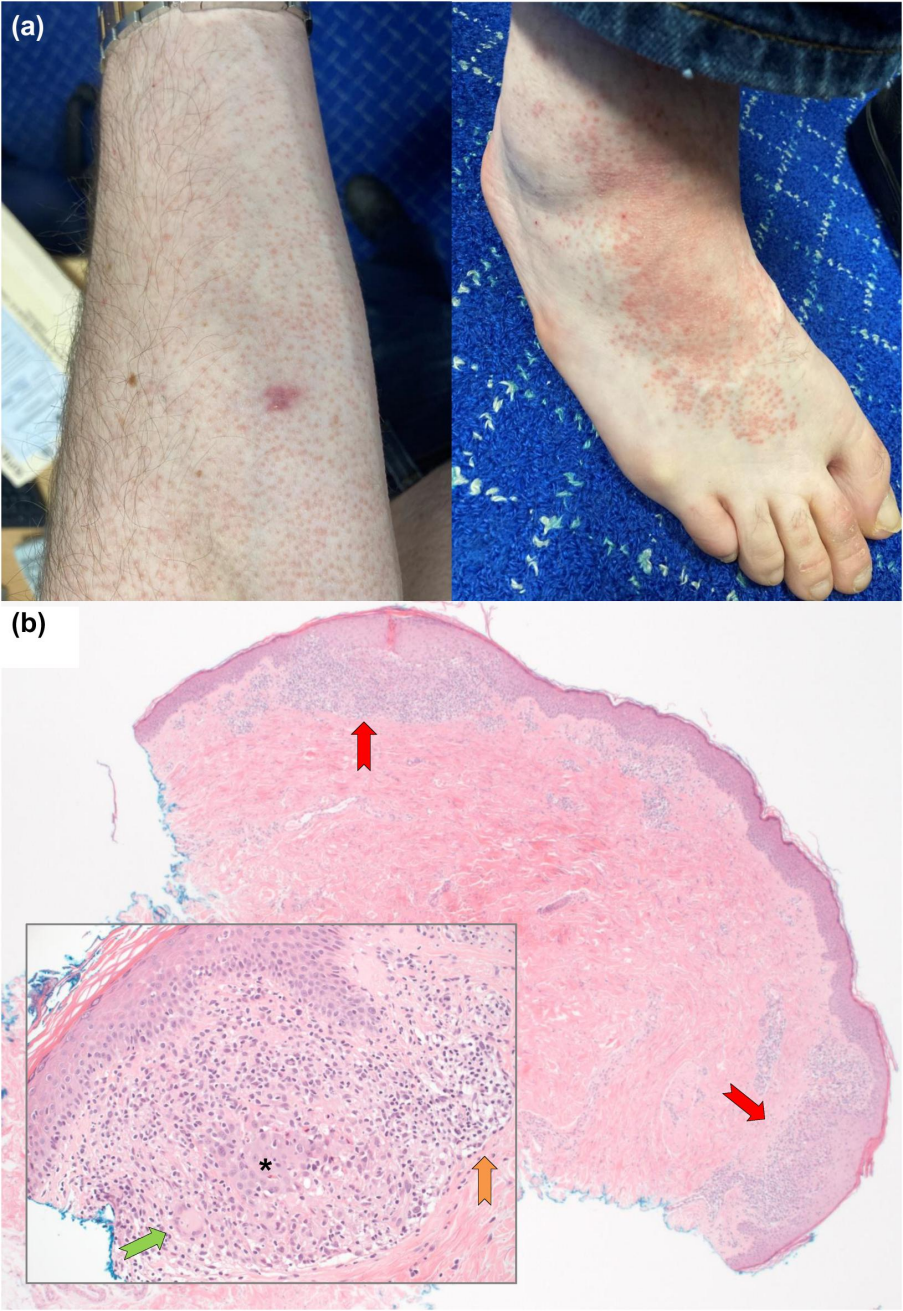
(a) Representative clinical image of cutaneous sarcoidosis, with orange‐red micropapules over the forearm (left image) coalescing into plaques on the dorsal foot (right image), April 2021; and (b) corresponding pathology (haematoxylin and eosin stain) displaying well‐formed, non‐caseating granulomas in superficial papillary dermis at X25 magnification [red arrows], consisting of (inset at X250 magnification) epithelioid histiocytes [*], occasional Langhan's‐type multinucleated giant cells [green arrow] and peripheral lymphocytic inflammation [orange arrow]. Special stains for fungi and mycobacteria were negative

**FIGURE 2:**
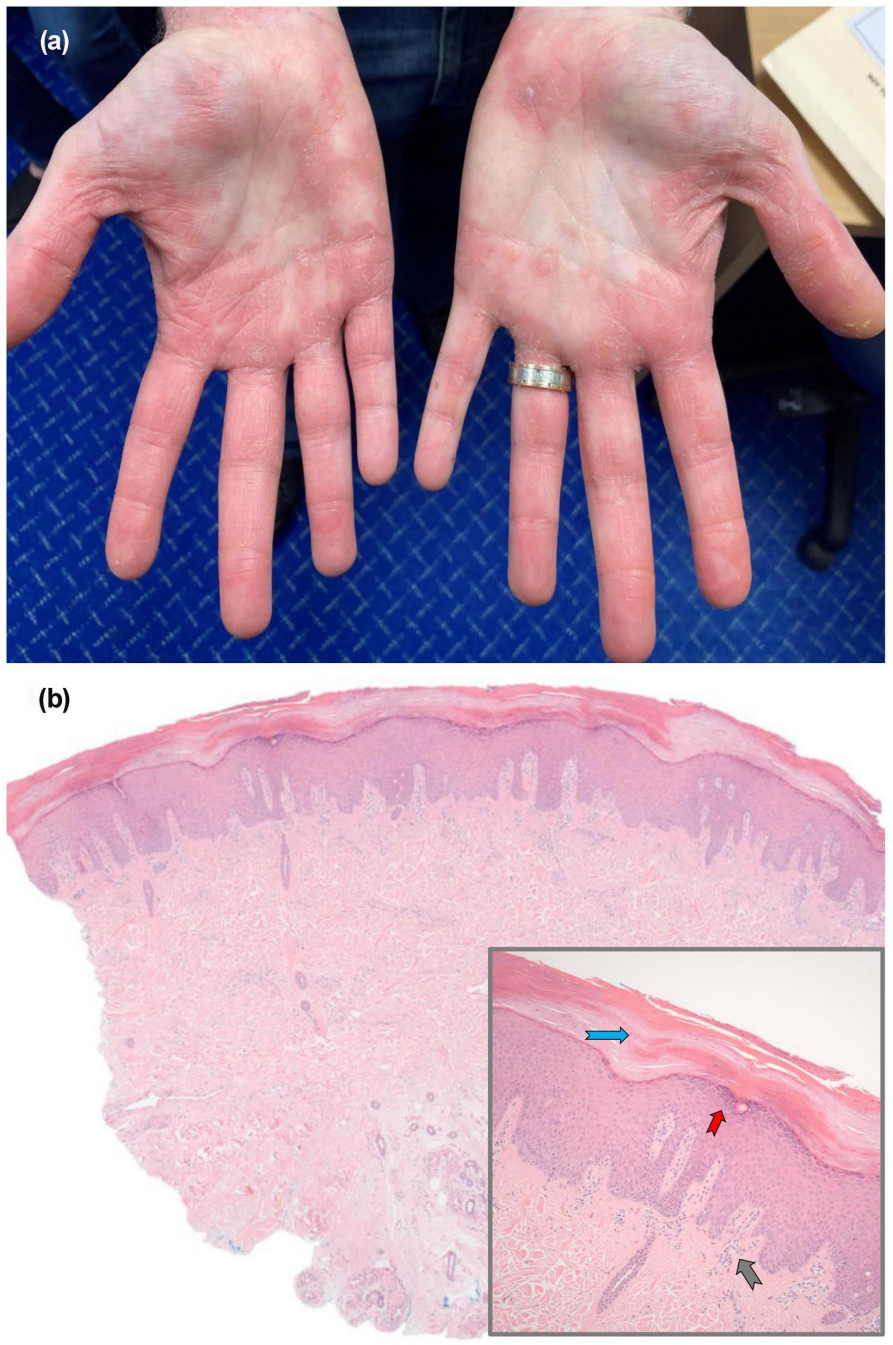
(a) Representative clinical image of orange‐red palmar keratoderma (psoriasiform/PRP‐like), April 2021; and (b) corresponding pathology (haematoxylin and eosin stain) showing compact acanthosis and hyperkeratosis, at X25 magnification, with (inset at X250 magnification) patchy checkerboard parakeratosis [blue arrow], variable hypergranulosis [red arrow] to hypogranulosis, and sparse lymphocytic infiltrate in the superficial papillary dermis [grey arrow]

In April 2021 the micropapular eruption extended to involve his torso, limbs, and dorsal feet (Figure 1a). Concurrently, he had also developed pruritic, orange‐red psoriasiform palmar plaques (Figure 2a). Biopsy of the micropapules revealed non‐caseating granulomas of epithelioid histiocytes and multinucleated giant cells in the superficial dermis, again consistent with cutaneous sarcoidosis (Figure 1b). Biopsy of the palmar plaques revealed a hyperkeratotic and acanthotic epidermis with checkerboard parakeratosis and variable hypo‐to‐hypergranulosis (Figure 2b); – resembling a psoriasiform/pityriasis rubra pilaris (PRP)‐like palmar keratoderma. On this occasion serum ACE was within normal limits, there was no lymphadenopathy or pulmonary infiltrates on chest computed tomography, and multi‐system reviews were again unremarkable.

Application of topical steroids was impractical for the patient, so hydroxychloroquine was initiated. In June 2021 progressive lower limb cutaneous metastases triggered change from the novel *BRAF* inhibitor to another clinical trial of combined *MEK*/*FAK* inhibitors. On review two months later, the micropapular sarcoid and palmar plaques had completely resolved.

**FIGURE 3:**
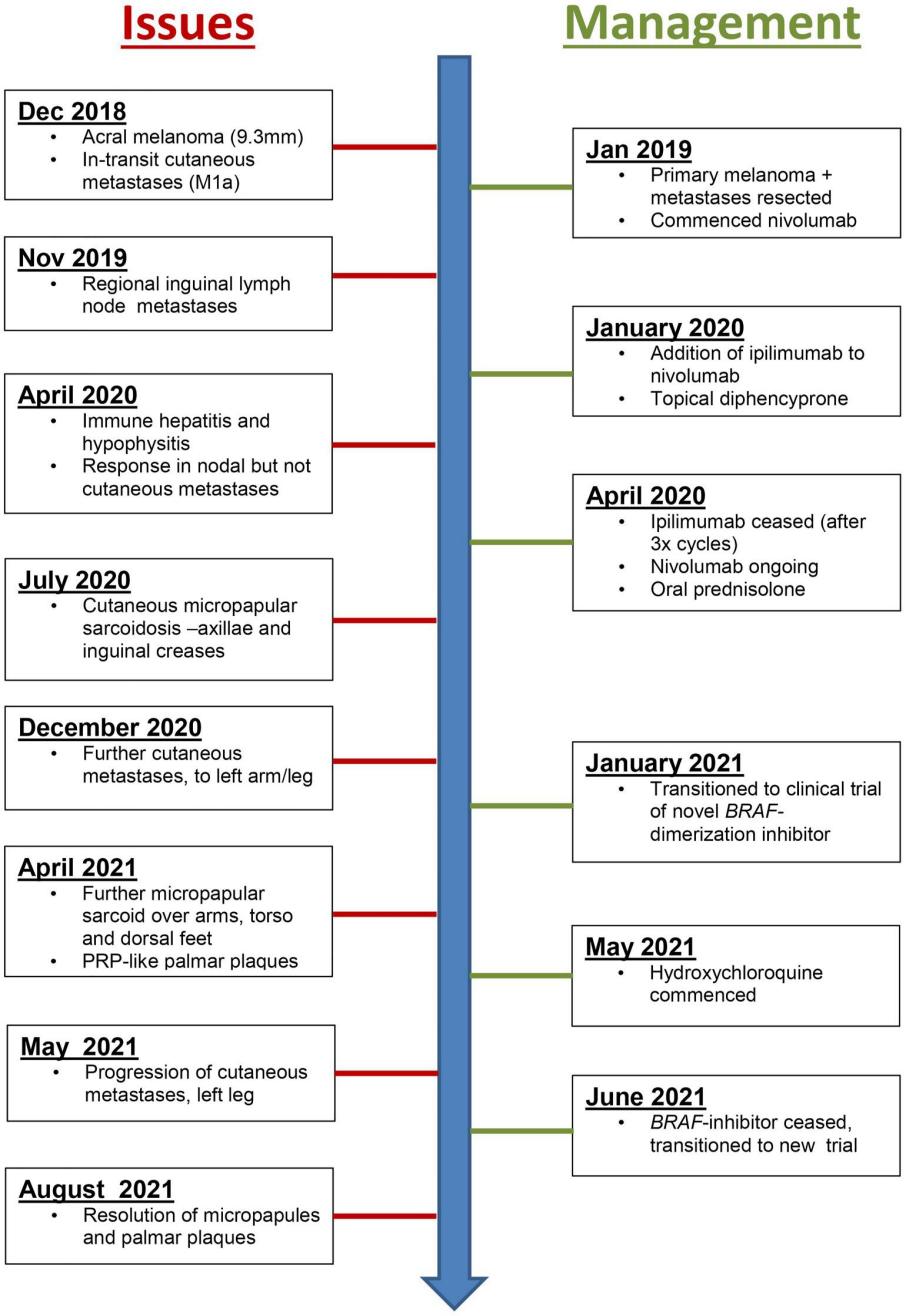
Timeline of pertinent medical issues and management changes, from December 2018 to August 2021

A timeline of medical issues and management changes are summarised in Figure 3.

## 4 DISCUSSION

Sarcoidosis is a non‐infectious granulomatous inflammatory disorder of unknown aetiology, although upregulation of T_H_1 and T_H_17 pathways are hypothesised.^3^ Systemic involvement can include pulmonary, nodal, ocular or cardiac sarcoidosis; with serum ACE frequently elevated.^3^ 25%–30% of patients with sarcoidosis have cutaneous involvement – commonly manifesting as erythematoviolaceous papules and plaques, or infiltration of tattoos and scars; with micropapular sarcoid a rare variant.^3^, ^4^

Nivolumab is an IgG_4_ monoclonal antibody binding to and inhibiting programmed cell‐death 1 (PD‐1) receptors, while ipilimumab is an IgG_1_ antibody inhibiting cytotoxic T‐lymphocyte‐associated protein 4 (CTLA‐4) receptors. Nivolumab and ipilimumab are immune‐checkpoint inhibitors (ICI's) blocking inhibitory or exhaustion signals on cytotoxic CD8+ T‐cells to direct immune activity against cancer cells, with superior outcomes compared to chemotherapy.^5^ Such increased immune activity can trigger immune‐related adverse events (irAE's) including sarcoidosis – with 22% of ICI‐treated patients in one cohort developing sarcoid‐like reactions.^1^, ^6^

Mechanisms of granuloma formation on ICI's are unclear, but may involve up‐regulation of T_H_17 T‐cell activity. CTLA‐4 inhibitors are associated with expansion of T_H_17 cells, while PD‐1 inhibitors increase their production of T_H_17 cytokines.^1^ ICI's can often be continued, with topical or systemic steroids effective in treating the sarcoidosis.^1^ While some irAE's are associated with superior survival outcomes on ICI therapy,^7^ it is unknown if this includes sarcoidosis.

Sarcoidosis has similarly been reported on *BRAF*‐inhibition.^2^ Driver mutations in the mitogen‐associated protein kinase (MAPK) pathway, including *BRAF* or *NRAS*, increase cellular proliferation and survival – promoting oncogenesis.^8^ *NRAS*‐mutant tumours, as in our case, are not amenable to first‐generation *BRAF*‐inhibitors (such as vemurafenib or dabrafenib) as these preferentially inhibit *BRAF*‐*v600E* mutant cells. With the exception of mutant *BRAF‐v600E*, dimerisation is required for *RAF*‐kinase activation.^8^ Our patient was treated with a novel *BRAF‐*inhibitor capable of inhibiting both monomeric and dimeric *BRAF*‐mutant kinases, as well as *RAF* dimerisation itself. Therefore, by inhibiting *RAF* fusion, such next‐generation *BRAF*‐inhibitors function as ‘pan‐*RAF*’ inhibitors, and unlike first‐generation *BRAF*‐inhibitors can be used to treat MAPK‐mutant tumours other than *BRAF*‐*v600E* (e.g., *NRAS*) with trials underway.^8^

It is unclear how *BRAF*‐inhibition results in granuloma formation, however insight can be gleaned from biochemical studies of first‐generation agents. Vemurafenib and dabrafenib increase serum interferon (IFN)‐γ and tumour necrosis factor (TNF)‐α, T_H_1 cytokines instrumental for T‐cell and macrophage activation, and thereby granuloma formation, in sarcoidosis.^1^, ^9^ Vemurafenib also induces expression of chemokines integral for macrophage recruitment in sarcoidosis (including CCL2 and CCL5), through antagonism of aryl‐hydrocarbon receptors.^10^ Furthermore, an analogue of vemurafenib decreases regulatory T‐cell (T_reg_) function in the tumour microenvironment, with impaired T_reg_‐cells also implicated in the pathophysiology of sarcoidosis.^3^, ^11^ Further studies are needed to ascertain if similar mechanisms contribute to sarcoidosis with inhibition of *BRAF*‐dimerisation.

In this case, the sarcoidosis was likely triggered by the ICI's then exacerbated by the novel *BRAF*‐inhibitor. Sarcoidosis can rarely develop spontaneously in melanoma patients, with an incidence of 0.42%,^12^ though temporal association with the drugs in this patient at both episodes suggests they were triggering factors. While sustained irAE's such as sarcoidosis from ICI's persisting post‐cessation is possible,^1^ the exacerbation on the *BRAF*‐inhibitor that remained until it was ceased suggests it also contributed to the eruption – possibly ‘primed’ by the ICI's. It is unknown if the *MEK*/*FAK* inhibitors contributed to the resolution of his sarcoid, as this has not been reported.

PRP‐like palmar plaques have not been previously reported on *BRAF*‐inhibition. Psoriasiform sarcoidosis is unlikely as no granulomas were evident on biopsy. An exacerbation of the patient's pre‐existing psoriasis is also unlikely as definitive histological features of psoriasis were not seen. Inhibition of TNF‐α and IL (interleukin)‐17 have shown efficacy in treating PRP, suggesting T_H_1/T_H_17 cytokines are implicated in its aetiology, as with sarcoidosis.^13^ TNF‐α and IL‐17 are also expressed by sarcoidal granulomas,^3^ and so the palmar plaques may have represented a psoriasiform/PRP‐like reaction to the sarcoidosis.

At both episodes of his sarcoid, cancer treatment was continued as the metastatic melanoma was deemed the treatment priority. Systemic immunosuppressants, such as methotrexate or TNF‐α inhibitors, were avoided as treatment for the sarcoid given their potential to promote melanoma progression.^14^ Hydroxychloroquine was chosen by the Oncology team given it can also augment anti‐neoplastic therapies through autophagy inhibition (acknowledging the risk of exacerbating psoriasiform eruptions).^3^, ^4^, ^15^

## 5 CONCLUSION

Our case demonstrates that next‐generation *BRAF*‐dimerisation inhibitors can exacerbate cutaneous sarcoidosis when primed by immune‐checkpoint inhibition. Dermatologists may encounter such reactions more frequently as uptake of these novel agents increases for treating cancers beyond clinical trials. Further studies are essential in determining whether *BRAF*‐dimerisation inhibition can trigger sarcoidosis *de‐novo*; as well as identifying therapeutic approaches for the sarcoid in patients with underlying cancers where immunosuppression is not ideal.

## CONFLICTS OF INTEREST

A. M. Menzies has served on advisory boards for BMS, MSD, Novartis, Roche, Pierre‐Fabre, QBiotics. R. P. M. Saw has received honoraria for advisory board participation from MSD, Novartis and Qbiotics and speaking honoraria from BMS and Novartis.

## AUTHOR CONTRIBUTIONS

**J. P. Pham:** Data curation; Investigation; Writing – original draft; Writing – review & editing. **P. Star:** Conceptualization; Data curation; Investigation; Writing – original draft; Writing – review & editing. **S. Wong:** Conceptualization; Data curation; Investigation; Writing – original draft; Writing – review & editing. **D. L. Damian:** Conceptualization; Data curation; Investigation; Writing – original draft; Writing – review & editing. **R. P. M. Saw:** Conceptualization; Data curation; Investigation; Writing – original draft; Writing – review & editing. **M. J. Whitfeld:** Conceptualization; Data curation; Investigation; Writing – original draft; Writing – review & editing. **A. M. Menzies:** Conceptualization; Data curation; Investigation; Writing – original draft; Writing – review & editing. **A. M. Joshua:** Conceptualization; Data curation; Investigation; Writing – original draft; Writing – review & editing. **A. Smith:** Conceptualization; Formal analysis; Investigation; Supervision; Writing – original draft; Writing – review & editing.

## Data Availability

The data that support the findings of this study are available on request from the corresponding author. The data are not publicly available due to privacy or ethical restrictions.
